# If it is in the marrow, is it also in the blood? An analysis of 1,000 paired samples from patients with B-cell non-Hodgkin lymphoma

**DOI:** 10.1186/1471-2407-10-644

**Published:** 2010-11-24

**Authors:** Patrizia Mancuso, Angelica Calleri, Pierluigi Antoniotti, Jessica Quarna, Giancarlo Pruneri, Francesco Bertolini

**Affiliations:** 1 Laboratory of Hematology-Oncology, European Institute of Oncology, via Ripamonti 435, 20141 Milan, Italy; 2 Division of Pathology-Laboratory Medicine, European Institute of Oncology, via Ripamonti 435, 20141 Milan, Italy

## Abstract

**Background:**

Staging of B-cell non Hodgkin's lymphoma (NHL) routinely involves bone marrow (BM) examination by trephine biopsy (BM-TB). The evidence of disease in the BM-TB results in a clinical stage IV classification affecting therapeutic strategies for NHL patients. BM immunophenotyping by flow cytometry (FC) is also used, although its clinical value is still under debate.

**Methods:**

Using FC we analyzed 1,000 paired BM aspirates and peripheral blood (PB) samples from 591 NHL patients to investigate the concordance between BM and PB. B-lymphocytes were defined monoclonal when a ratio of 0.3 < κ/l > 3 was observed. Aberrant immunophenotypes present in the B-cell subpopulation were also investigated. BM-TB was also performed in 84.1% of samples (841/1000), and concordance between BM-TB and BM-FC was evaluated. Concordance was defined as the presence of a positive (in terms of disease detection) or negative result in both BM-FC and PB-FC or BM-TB and BM-FC.

**Results:**

Using FC, the overall concordance between BM and PB was 95%. Among the discordant cases (ie presence of neoplastic B-lymphocyte in the BM but under the sensibility of the technique in the PB) the most frequent diagnosis was Waldenstrom's macroglobulinemia (WM, accounting for 20.8% of all discordant cases). The expression of CXCR4, a receptor involved in B-cell trafficking and homing, was found to be down regulated in WM compared to other NHL types, thus suggesting a possible role of CXCR4 in WM cell homing in the BM. WM excluded, FC investigation of BM and PB in NHL patients gives overlapping information.

BM involvement was observed by FC in 38% of samples, and concordance between BM-FC and BM-TB was 85%.

**Conclusions:**

The finding that FC data from BM and PB samples overlap in NHL might have major implications for the design of future clinical studies and for patients' follow-up.

## Background

Bone marrow (BM) examination by trephine biopsy (BM-TB) is routinely performed during staging and follow-up of patients affected by B-cell non-Hodgkin lymphoma (NHL). BM disease results in a stage IV classification, and may affect therapeutic strategies [1]. Flow cytometry BM immunophenotyping (BM-FC) is used in adjunct to BM-TB, even though its clinical value is still under investigation [2]. In the present study, in addition to the BM aspirate, the peripheral blood (PB) was analyzed to investigate if malignant cells were restricted to BM or circulating in the blood.

Chemokine receptors are expressed by a variety of cells, including lymphoid cells, and mediate cell trafficking and homing. These receptors may also be involved in the migration and dissemination of NHL cells [3]. The stromal derived factor -1 (SDF-1, CXCL12) chemokine plays a crucial role in the retention of a variety of cells into BM niches through its receptor CXCR4 [3,4]. We investigated CXCR4 in different NHL subtypes to assess whether its expression correlates with differences in the frequency of NHL cells in the PB versus the BM.

## Methods

We retrospectively analyzed 1,000 paired BM aspirates and PB samples from 591 NHL patients (i.e. 1000 BM samples along with 1000 PB samples from the same day) consecutively collected in our Institute from 2000 to 2007. BM-TB was also performed in 84.1% of paired samples (841/1000), and, as BM-TB is considered the "gold-standard" for NHL staging and follow up, we also evaluated concordance between BM-TB and BM-FC.

Among the 1000 consecutive paired samples, 31% were collected at the time of first diagnosis (616/2000), and 69% after therapy. For all patients, the diagnosis of NHL was obtained by morphology, phenotype and molecular analysis of nodal or extra-nodal sites and established according to the World Health Organization recommendations [5]. Hairy Cell Leukaemia, T-cell NHL, Hodgkin disease and multiple myeloma patients were not included in this study. The different subtypes of B-cell NHL are described in Table 1.

**Table 1:** Patient's Characteristics

Diagnosis	Number of Patients	% of Patients
Follicular Lymphoma (FL)	205	35%

Diffuse Large B cell Lymphoma (DLBC-L)	142	24%

Mantle Cell Lymphoma (MCL)	47	8%

Marginal Zone Lymphoma - Mucose Associated Lymphoma Tissue (MZL-MALT)	74	13%

Lymphocytic lymphoma/chronic lymphocytic leukemia (CLL)	83	14%

Waldenstrom's macroglobulinemia (WM)	40	7%

TOTAL	591	100%

**Figure 1:**
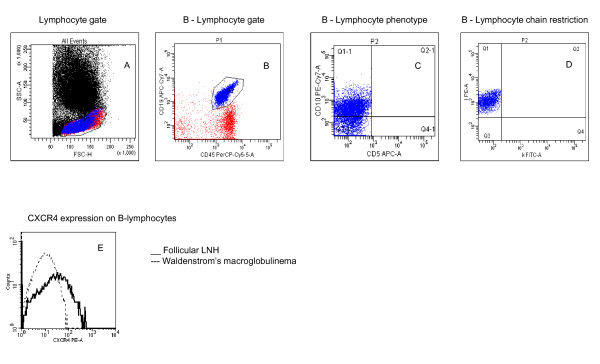
**A-D: Representative FC dot plots showing the sequential multiparametric detection of clonal restriction in B-Lymphocytes from a patient with follicular NHL**. Panel A shows the gate made on lymphocytes; Panel B shows CD19 and CD45 expression in the lymphocyte gate; Panel C shows the expression of CD5 and CD10; Panel D shows lambda chain clonal restriction; Panel E shows representative CXCR4 expression in patients with follicular LNH and WM.

Four- (or, after 2005, six-) colour multiparametric FC was performed (Figure 1). Monoclonal antibodies including anti-CD45, -CD19, -CD20, surface IgM, -CD10, -CD5, -CD43, -CD23, anti-κ and anti -λ Ig light chain were used to analyze the B-lymphocyte immunophenotype. When light chain restriction was observed, anti-CD38, FMC-7, CD79b, CD22, CD103, CD11c, CD25 expression were also investigated on B-cells to better characterize B-cell phenotype [6] and to investigate the concordance between the diagnosis reported and the phenotype of the pathological B-lymphocytes observed. To detect light chain restriction, we used anti-κ FITC/anti -λ Pe/CD45 PerCP/CD19 APC or anti-κ FITC/anti -λ Pe/CD45 PerCP/CD10 Pe-Cy-7/CD5 APC/CD19 APC-Cy-7. As CD19-APC-Cy7 shows a very low signal-to-noise ratio in some cases like FL/DLBCL with low level CD19, the combination CD19 Pe/CD45 PerCP was used to compare the percentage of CD19 positive cells obtained with both markers. B-lymphocytes were defined monoclonal when a ratio of 0.3 < κ/l > 3 was observed [7] or, for some lymphocytic lymphoma/chronic lymphocytic leukaemia (CLL) patients, when surface membrane light chains were absent. At least 100 CD19+ events showing the expected immunophenotype were required to define a FC test as positive [8]. In addition to this panel, anti-CD3, -CD4, -CD8 and -CD16+56 were routinely analyzed to gain information regarding the distribution of lymphocyte sub-populations. Controls included substitution of the relevant monoclonal antibody by a murine Ig of the same isotype. Monoclonal antibodies were purchased from BD Biosciences (Mountain View, CA), and used at the recommended concentration.

We also evaluated CXCR4 expression on B-cells in 54 paired BM-aspirates and PB (10 WM, 14 DLBC, 18 FL and 12 CLL).

For CXCR4 detection, 10 μl of PE-conjugated anti CXCR4 (BD, clone 12g5) was used in combination with anti CD45 Per-CP and CD19 Pe-Cy7. An isotype-matched negative control was used. Mean fluorescence intensity (MFI) ratios were calculated by dividing the MFI for CXCR4 by the MFI of the respective isotype-matched negative control, both in the BM aspirates and in PB. For sample preparation, a stain - lyse and wash standard procedure was used. Red cells were lysed by a NH_4_Cl solution. Measurements were performed on a FACS-Calibur or FACS-Canto (BD Biosciences), equipped with a second laser. Acquisition was stopped after collection of more than 200.000 total events or when at least 100 CD19 positive events were collected. Sensitivity of the procedure was established by serial dilution experiments as 1/10,000.

BM-TB were fixed in 10% buffered formalin, decalcified with EDTA, embedded in paraffin and stained with haematoxylin-eosin. Immunohistochemistry with anti CD3-, CD5, CD10, CD20, CD23, CD34, CD43, CD79a, bcl-2, bcl-6, cyclin D1, Ki-67 was performed to distinguish between reactive and neoplastic lymphoid infiltration and to differentiate the different subtypes.

Concordance was defined as the presence of a positive (in terms of disease detection) or negative result in both BM-FC and PB-FC or BM-TB and BM-FC. Statistical comparisons were performed using the t-test, analysis of variance (ANOVA) and linear regression when data were normally distributed and the non-parametric analyses of Spearman and Mann-Whitney when data were not normally distributed. Values of p lower than 0.05 were considered as significant.

## Results

### Concordance between BM-FC and PB-FC

As shown in Table 2, the most frequent diagnoses were Follicular NHL (FL, 35%) and Diffuse Large B-Cell NHL (DLBC, 24%). The overall FC concordance between BM and PB was 95.2% (Table 2) in all NHL subtypes, and the sensitivity of BM-FC was higher than that of PB-FC. In all discordant cases (4.8%), monoclonal B lymphocytes were present in the BM, whereas in PB the monoclonal cell population was absent or under the sensitivity of the procedure. This was observed at diagnosis and after CT (p < 0.001 in both cases). In discordant cases, BM monoclonal B-cells ranged from 0.06% to 18% of all leucocytes. Among the 96 discordant cases, the most frequent diagnosis was Waldenstrom's macroglobulinemia (WM) (20.8%). Sixty-two percent of discordant samples were collected after chemotherapy, and in discordant samples the most frequent treatment (38.5% of all discordant cases) was Rituximab alone or in association with chemotherapy.

**Table 2:** Concordance between BM-FC and PB-FC

		Pts		Samples		BM+PB+		BM+PB-		BM-PB+	BM-PB-	
		
		N°	%	N°	%	N°	%	N°	%	N°	N°	%
FL	diag			250	13%	78	31,2%	12	4,8%	0	160	64,0%
	
	after CT			454	23%	68	15,0%	14	3,1%	0	372	81,9%
	
	both	205	35%	704	35%	146	20,7%	26	3,7%	0	532	75,6%

DLBC	diag			146	7%	22	15,1%	10	6,8%	0	114	78,1%
	
	after CT			218	11%	56	25,7%	4	1,8%	0	158	72,5%
	
	both	142	24%	364	18%	78	21,4%	14	3,8%	0	272	74,7%

MCL	diag			24	1%	21	87,5%	1	4,2%	0	2	8,3%
	
	after CT			198	10%	69	34,2%	1	0,5%	0	128	63,4%
	
	both	47	8%	222	11%	90	39,8%	2	0,9%	0	130	57,5%

MZL-MALT	diag			74	4%	26	35,1%	3	4,1%	0	45	60,8%
	
	after CT			154	8%	56	37,3%	2	1,3%	0	96	64,0%
	
	both	74	13%	228	11%	82	36,6%	5	2,2%	0	141	62,9%

CLL	diag			78	4%	74	94,9%	0	0,0%	0	4	5,1%
	
	after CT			236	12%	152	64,4%	14	5,9%	0	70	29,7%
	
	both	83	14%	314	16%	226	72,0%	14	4,5%	0	74	23,6%

WM	diag			44	2%	23	52,3%	10	22,7%	0	11	25,0%
	
	after CT			124	6%	25	20,2%	25	20,2%	0	74	59,7%
	
	both	40	7%	168	8%	48	28,6%	35	20,8%	0	85	50,6%

TOTAL		591	100%	2000	100%	670	33,5%	96	4,8%	0	1234	61,7%

### Concordance between BM-TB and BM-FC

Results of BM-TB are reported in Table 3. The concordance between BM-FC and BM-TB was 84% (83% at diagnosis and 85% after treatment). This result is in line with previous studies dealing with smaller series of patients that showed a 79-90% concordance [9].

**Table 3:** BM-TB results

		Samples		BM+		BM-	
		
		N°	%	N°	%	N°	%
FL	diag	96	31%	32	42%	64	28%
	
	after CT	212	69%	44	58%	168	72%
	
	both	308	36%	76	25%	232	75%

DLBC	diag	68	41%	9	13%	59	87%
	
	after CT	99	59%	12	12%	87	88%
	
	both	167	20%	21	13%	146	87%

MCL	diag	12	13%	7	58%	5	42%
	
	after CT	78	87%	28	36%	50	64%
	
	both	90	11%	35	39%	55	61%

MZL-MALT	diag	33	32%	11	33%	22	67%
	
	after CT	69	68%	16	23%	53	77%
	
	both	102	12%	27	26%	75	74%

CLL	diag	23	20%	20	87%	3	13%
	
	after CT	90	80%	59	66%	31	34%
	
	both	113	13%	79	70%	34	30%

WM	diag	15	25%	13	87%	2	13%
	
	after CT	46	75%	34	74%	12	26%
	
	both	61	7%	47	77%	14	23%

TOTAL		841	100%	285	34%	556	66%

**Figure 2:**
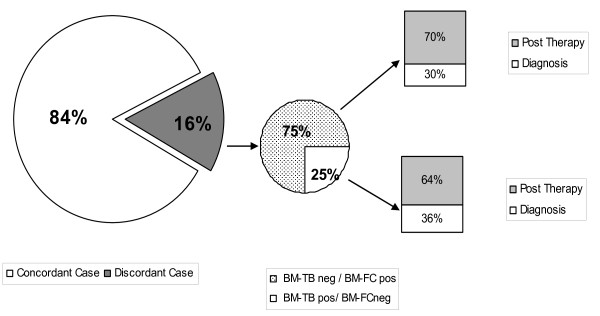
**Analysis of discordance between BM-FC and BM-TB**.

Among discordant cases (16%, 135 samples, Figure 2), 75% of the samples (101 samples) were BM-FCpos/BM-TBneg and 25% (34 samples) were BM-FCneg/BM-TBpos. Among BM-FCpos samples, 30% (30 samples) were evaluated at first diagnosis (vs 36%, 12 samples, of BM-TPpos cases at first diagnosis, p = 0.79) and 70% (71 samples) were evaluated after therapy (vs 64%, 22 samples, of BM-TBpos cases after therapy, p = 0.89).

In BM-FCpos/BM-TBneg samples, the percentage of monoclonal B cells detected by FC was lower than 1% of all leukocytes, while in BM-FCneg/BM-TBpos samples, the most frequent finding was the presence of a nodular infiltrate. Differences in the frequency of positivity in the BM investigated by FC or TB were generally not significant, with the exceptions of MZL-MALT after therapy (38.6% by FC vs 23% by TB, p = 0.036), and WM after therapy (40.4% by FC vs 74% by TB, p < 0.001). The difference observed in MCL at diagnosis was of borderline significance (91.7% by FC vs 58% by TB, p = 0.053). At diagnosis, BM-TBneg/BM-FCpos cases were 0, 8, 33, 6, 8, and 0% in FL, DLBC, MCL, MZL-MALT, CLL and WM, respectively. After CT, BM-TBneg/BM-FCpos cases were 0, 14, 0, 15, 4, and 0% in FL, DLBC, MCL, MZL-MALT, CLL and WM, respectively.

### CXCR4 expression in different NHL subtypes

In WM, we observed a significant CXCR4 down-regulation in comparison to other NHL types (Figure 1). Median MFI ratio was 6 in WM vs 15, 10 and 34 in DLBC-L, FL and CLL, respectively (p < 0.01).

CXR4 expression in B lymphocytes in the BM and in the PB was not significantly different (data not shown). The crucial role of CXCR4 in WM cell homing in the marrow has been recently described [10]. Our current results suggest that CXCR4 expression is variable among different NHL subtypes, and lead to speculate a CXCR4 involvement in NHL blood spreading, possibly explaining the discordant results of BM and PB analyses in WM versus other NHL types. In fact, a lower CXCR4 MFI ratio was found in diseases where the presence of circulating neoplastic B cells is less frequent (WM and DLBC), and a higher ratio was found in NHL types associated with the presence of circulating neoplastic B cells in a larger frequency of cases (FL and CLL). Further studies are now needed to validate this hypothesis.

## Discussion

BM examination by BM-TB is an integral part of the clinical staging and follow up of NHL patients. Along with BM-TB, BM-FC is used as an ancillary investigation, even though its use is still debated. The correlation between these two techniques was found to be very high, and our results are in line with previous studies dealing with smaller series of patients (85% vs 80%-90%, see ref. [9]).

However, as BM aspiration might be a painful procedure for patients, we retrospectively analyzed 1,000 paired BM aspirates and PB samples from NHL patients to assess if PB analysis could be an alternative reliable procedure for staging and follow up of these patients. The overall FC concordance between BM and PB was 95.2%. Among the discordant cases, 62% of samples were collected after chemotherapy and the most frequent treatment was Rituximab alone or in association with chemotherapy. This result suggests that during treatment with Rituximab alone or in association with chemotherapy, neoplastic B cells may be depleted in the PB but still present in the BM. Therefore, a negative PB sample obtained during treatment with Rituximab should be considered with caution [8].

Among discordant cases, the most frequent diagnosis was WM (20.8%). Among the different biological reasons that might explain this discrepancy, we focused on CXCR4. The crucial role of CXCR4 in WM cell homing in the BM has been recently described [10] and our results, even though obtained in a small number of patients and samples, confirm this hypothesis. In fact, CXCR4 expression on B lymphocytes was found to be down-regulated when compared to other NHL types (p < 0.001).

Albeit the investigation of NHL patients' clinical outcome was beyond the scope of the present study, encompassing a large variety of NHL types and treatment plans, we tried to investigate the clinical outcome of the discordant cases. However, the clinical follow-up of the discordant cases did not offer any meaningful and significant information, because of the very large variety of different treatment procedures in the patients.

To the best of our knowledge, only two previous papers have reported about the role of FC in clinical NHL outcome. Perea *et al *[9] reported discordance between BM-FC and BM-TB (BM-FCpos/BM-TBneg) in 9% (36) of low-grade NHL patients, in most cases (66%) affected by FL. In this study, discordance had no apparent influence on the clinical outcome when compared with BM-negative patients (median follow-up 14 months). In the second study, Gronich *et al *[11] investigated 70 low-grade NHL patients and reported that the outcome in patients who had BM involvement defined by FC alone or by morphology was similar.

## Conclusions

Our data indicate that - WM excluded - FC investigation of BM and PB in NHL patients gives overlapping information. This finding might have major implications for the design of future NHL clinical studies and suggest that the FC investigation of PB has potential as a possible alternative to BM-TB for the follow-up of NHL patients.

## Competing interests

The authors declare that they have no competing interests.

## Authors' contributions

PM, GP, and FB: designed the research, analyzed the data, and wrote the paper.

AC, PA, JQ performed flow cytometric analysis, and analyzed the data. All authors read and approved the final manuscript.

## Pre-publication history

The pre-publication history for this paper can be accessed here:

http://www.biomedcentral.com/1471-2407/10/644/prepub
